# Population-based estimate of hepatitis C virus prevalence in Ontario, Canada

**DOI:** 10.1371/journal.pone.0191184

**Published:** 2018-01-23

**Authors:** Shelly Bolotin, Jordan J. Feld, Gary Garber, William W. L. Wong, Fiona M. Guerra, Tony Mazzulli

**Affiliations:** 1 Public Health Ontario, Toronto, Ontario, Canada; 2 Dalla Lana School of Public Health, University of Toronto, Toronto, Ontario, Canada; 3 Toronto Centre for Liver Disease, Toronto General Hospital, Toronto, Ontario, Canada; 4 Sandra Rotman Centre for Global Health, University of Toronto, Toronto, Ontario, Canada; 5 Ottawa Hospital Research Institute, Ottawa, Ontario, Canada; 6 School of Pharmacy, University of Waterloo, Kitchener, Ontario, Canada; 7 Mount Sinai Hospital/University Health Network and Department of Laboratory Medicine and Pathobiology, University of Toronto, Toronto, Ontario, Canada; Centers for Disease Control and Prevention, UNITED STATES

## Abstract

**Background:**

Hepatitis C virus (HCV) is the most burdensome infectious illness in Canada. Current screening strategies miss a significant proportion of cases, leaving many undiagnosed. Elevated HCV prevalence in those born between 1945 and 1965 has prompted calls for birth-cohort screening in this group. However, Canada lacks population-level data to support this recommendation. We performed a serosurvey to obtain population-based HCV prevalence estimates in Ontario residents born between 1945–1974, to generate evidence for birth-cohort screening recommendations.

**Methods:**

We tested anonymized residual sera in five-year age-sex bands from Ontario for anti-HCV antibody. We performed descriptive epidemiological analysis and used a logistic regression model to determine HCV risk-factors.

**Results:**

Of 10,006 sera analyzed, 155 (1.55%, 95% confidence interval (CI) 1.32, 1.81) were positive for HCV antibody. Individuals born between 1950–1964 had a significantly higher combined prevalence of 1.92% (95% CI 1.56, 2.34) compared to 1.14% (95% CI 0.69, 1.77) (p = 0.04) for those born between 1970–1974. For males, comprising 107/155 (69.03%) of positive samples, the highest prevalence was 3.00% (95% CI 1.95, 4.39) for the 1960–1964 birth-cohort. For females, the highest prevalence was 1.56% (95% CI 0.83, 2.65) for those born between 1955–1959. Male sex was significantly associated with positive HCV serostatus.

**Interpretation:**

HCV prevalence in Ontario is highest among those in this birth cohort, and higher than previous estimates. The prevalence estimates presented in our study provide important data to underpin birth-cohort screening recommendations.

## Introduction

Infection with hepatitis C virus (HCV) is a growing public health concern globally, with 130–150 million chronic cases worldwide and 700,000 deaths annually from HCV-related liver disease [1]. In Canada, HCV is estimated using modeling to chronically infect between 220,697 and 245,987 individuals [2] and causes the greatest burden of illness of any infectious disease in the country [3]. The majority of those acutely infected are unable to clear the virus, resulting in chronic infection which can progress to cirrhosis and its complications, including hepatocellular carcinoma and liver failure [4].

HCV testing guidelines have historically been directed at patients in high-risk groups, including persons who inject drugs, incarcerated individuals [5], symptomatic individuals or those with evidence of chronic liver disease [6]. However, targeted testing often misses a significant proportion of the infected population. Persons in many of the high-risk groups are less likely to access healthcare [7], and once in care must be recognized by physicians as high-risk to prompt testing. Many infected patients are unaware of their risk factors or choose not to report them because of the stigma associated with high-risk behaviors [8–11]. Symptom-based screening also results in incomplete case finding because most patients have few or no symptoms until liver damage is very advanced [4]. Targeted screening has therefore left a significant proportion of the infected population in Canada undiagnosed. Although precise data are lacking, a recent modeling study suggests that only 56% of HCV-infected individuals in Canada have been diagnosed [2]. The Canadian Health Measures Survey found that only 31% were aware of their infection [11]. However, there are many uncertainties around these figures, partially stemming from poor estimates of national prevalence [12]. Under-diagnosis of HCV is particularly alarming given the rapid progress in development of highly effective well-tolerated antiviral therapy which can cure the infection in upwards of 95% of those treated [13].

Recent evidence shows that HCV-associated morbidity and mortality is rising [10,14], particularly in the birth cohort born between 1945 and 1965 [9,10,15]. To address this, the Centres for Disease Control and Prevention (CDC) advocated for one-time birth cohort screening for those born between 1945 and 1965, citing evidence that this would identify over 75% of infected individuals and would be cost-effective by preventing downstream complications of HCV infection [10]. Birth cohort screening has also been suggested in Canada, where approximately three-quarters of current cases were born between 1945 and 1975 [8,16]. The cost-effectiveness of this strategy has been demonstrated [17], and has been recommended by the Canadian Liver Foundation for those born between 1945–1975, [18] and others [8]. However, recently published guidelines from the Canadian Task Force on Preventative Health Care have recommended against screening asymptomatic adults, including the birth-cohort born between 1945–1975 [19].

To date, national prevalence estimates for HCV in Canada are based largely on modeling studies, which relied on limited and often poor quality, non-population level data, making it very difficult to develop appropriate policy recommendations. The aim of this study was to perform a serosurvey to obtain a population-based estimate of the prevalence of HCV infection in Ontario by birth cohort, allowing for an estimate of the number of HCV cases by age cohort in Canada.

## Materials and methods

### Selection of study population

**Fig 1:**
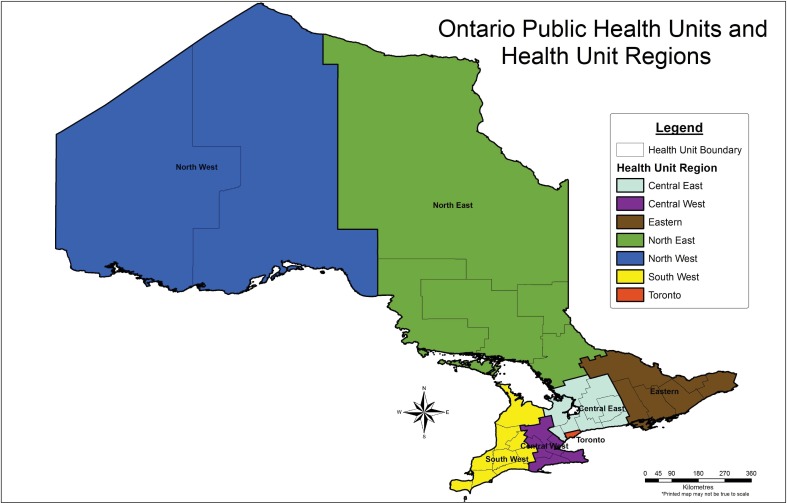
Ontario public health units and health unit regions.

Based on the Canadian Liver Foundation screening recommendations and cost-effectiveness studies, we included residents of Ontario born between 1945 and 1974 [17,18] with a similar number of males and females included in each five-year age-sex band. To account for differences in population densities and possible geographic variability in prevalence across Ontario, the number of sera in the study originating from each of seven public health regions in Ontario varied according to population density in each region, ensuring that our sample was geographically representative of the population of Ontario (Fig 1) [20].

### Sample size calculations

Sample size calculations, performed in Stata 12 (Stata Corporation, College Station, Texas), showed that approximately 10,000 sera, or approximately 833–835 sera per five-year age-sex band, would be required to provide a seroprevalence estimate that would be precise to ±0.2%, ±0.3% and ±0.35% for a prevalence estimate of 1%, 2% and 3%, respectively.

### Sample collection

We obtained anonymized residual sera from the largest private diagnostic laboratory in Ontario (LifeLabs, Mississauga, Ontario, Canada). At this laboratory, the most commonly performed tests using sera include electrolytes, lipids and glucose tests. In Ontario, private diagnostic laboratories perform much of the testing for primary care physicians. Through this approach we therefore attempted to target a healthier outpatient population compared to acute or chronically ill populations that are found in hospitals, or samples that required reference testing (including confirmatory testing for HCV), which are commonly submitted to the Public Health Ontario (PHO) Laboratory. Samples in the required strata were collected from August 2014 to February 2015. Following required diagnostic testing, serum samples were de-identified with only information on sex, year-band and Ontario region remaining. Any serum specimen collected from an individual within the birth cohort of interest was eligible for inclusion, provided that it was collected in a serum separator tube, contained a minimum of 2 mL of residual serum and was shipped to PHO within 48 hours of collection.

### Laboratory testing

Samples were shipped to PHO and tested immediately upon arrival. Sera were initially screened for HCV antibody using the Architect Anti-HCV assay (Abbott Diagnostics, Abbott Park, IL, USA). All positive sera were then tested using the Siemens Anti-HCV assay (Siemens Healthcare, Erlangen, Germany) for confirmation.

### Statistical analyses

A univariate analysis of the survey sample, including the proportion from each sex, age-band and region was performed. A bivariate analysis was used to describe these characteristics for HCV antibody positive samples and Wald chi-square tests were used for comparison between groups. Seroprevalence estimates and exact 95% confidence intervals (CI) were calculated for each age-band, sex, age-sex band and region in Ontario. Logistic regression was performed using a forward-building strategy. First, univariate logistic regression was used with HCV antibody positivity as the dependent variable, and sex, age-band and region separately as independent variables. A multivariable logistic regression model was then completed including all independent variables. A test for interaction was performed between age-band and sex.

When comparing prevalence between year-bands of birth, we used the 1970–1974 age-band as a reference, because although this year-band of birth is included in the Canadian Liver Foundation screening recommendation, it is not thought to be part of the birth cohort with elevated HCV prevalence, and jurisidictions such as the US have shown that the prevalence in this group is markedly lower than older cohorts included in our study [10]. To extrapolate estimates to the Canadian population, the age structure was based on 2016 census data [21] and estimates of HCV prevalence outside of the tested cohort were based on modeling data from Remis et al. [16].

### Ethics

Ethics approval for this study was granted by the Public Health Ontario Ethics Review Board. Samples were de-identified prior to HCV testing and thus we were unable to recontact any patients positive for HCV to inform them of their status.

## Results

### Risk factors for HCV antibody prevalence

In total, 10,006 sera were included in the analysis (Table 1). Each five-year band comprised between 1666–1669 specimens, representing approximately 16.7% of the total study sample. Overall, 155/10,006 (1.55%, 95% CI 1.32, 1.81) samples were positive for HCV antibody. Seroprevalence estimates varied by year-band of birth (p = 0.08) (Table 2). The oldest and youngest year-bands had the lowest proportions of samples with HCV antibody, with a seroprevalence of 1.02% (95% CI 0.60, 1.63) for samples from individuals born between 1945–1949 and a seroprevalence of 1.14% (95% CI 0.69, 1.77) for samples from individuals born between 1970–1974. Sera from individuals born between 1950–1954 and 1960–1964 had the highest seroprevalence, each at 1.98% (95% CI 1.37, 2.77). Sera from individuals born between 1950–1964 comprised 61.9% of all HCV antibody positive samples, and had a combined prevalence of 1.92% (95% CI 1.56, 2.34). This was significantly higher than the prevalence of our reference group, which was the youngest cohort, born between 1970–1974, which had a prevalence of 1.14% (95% CI 0.69, 1.77) (p = 0.04). Sera from individuals born between 1950–1969 comprised 76.8% of all antibody positive samples, with a combined prevalence of 1.78%, (95% CI 1.48, 2.13). Although the prevalence in this cohort was elevated compared to other age-bands, the difference was not statistically significant when comparing to those born between 1970–1974 (p = 0.07).

**Table 1:** Residual sera tested for HCV antibody by birth cohort year-band and geographic region, Ontario 2014–2015.

Year-band	Samples from each region							Total N (%)
	Central East N (%)	Central west N (%)	Eastern N (%)	North West N (%)	North East N (%)	South West N (%)	Toronto N (%)	
**1945–49**	466 (27.97)	319 (19.15)	241 (14.47)	30 (1.80)	88 (5.28)	219 (13.15)	303 (18.19)	1666 (100.00)
**1950–54**	475 (28.49)	315 (18.90)	232 (13.92)	34 (2.04)	85 (5.10)	215 (12.90)	311 (18.66)	1667 (100.00)
**1955–59**	485 (29.08)	315 (18.88)	229 (13.73)	34 (2.04)	82 (4.92)	209 (12.53)	314 (18.82)	1668 (100.00)
**1960–64**	506 (30.34)	319 (19.12)	223 (13.37)	30 (1.80)	76 (4.56)	197 (11.81)	317 (19.00)	1668 (100.00)
**1965–69**	515 (30.86)	320 (19.17)	210 (12.58)	28 (1.68)	64 (3.83)	181 (10.84)	351 (21.03)	1669 (100.00)
**1970–74**	496 (29.74)	319 (19.12)	205 (12.29)	26 (1.56)	60 (3.60)	178 (10.67)	384 (23.02)	1668 (100.00)
**Total**	**2,943 (29.41)**	**1,907 (19.06)**	**1,340 (13.39)**	**182 (1.82)**	**455 (4.55)**	**1,199 (11.98)**	**1,980 (19.79)**	**10,006 (100.00)**

**Table 2:** Demographic characteristics of individuals with HCV seropositive sera, Ontario 2014–2015.

Characteristic	Total samples tested (N = 10006) n	HCV antibody status		
		Total HCV seropositive (N = 155) n	Seroprevalence % (95% CI)	Chi square p-value
**Year-band**				
**1945–1949** **1950–1954** **1955–1959** **1960–1964** **1965–1969** **1970–1974**	1666 1667 1668 1668 1669 1668	17 33 30 33 23 19	1.02 (0.60, 1.63) 1.98 (1.37, 2.77) 1.80 (1.22, 2.56) 1.98 (1.37, 2.77) 1.38 (0.88, 2.06) 1.14 (0.69, 1.77)	0.08
**Sex**				
**Male** **Female**	5003 5003	107 48	2.14 (1.76, 2.58) 0.96 (0.71, 1.27)	<0.0001
**Region**				
**Central East** **Central West** **Eastern** **North East** **North West** **South West** **Toronto**	2943 1907 1340 455 182 1199 1980	44 27 19 4 4 21 36	1.50 (1.09, 2.00) 1.42 (0.94, 2.05) 1.42 (0.86, 2.21) 0.88 (0.24, 2.24) 2.20 (0.60, 5.53) 1.75 (1.09, 2.66) 1.82 (1.28, 2.51)	0.75

**Fig 2:**
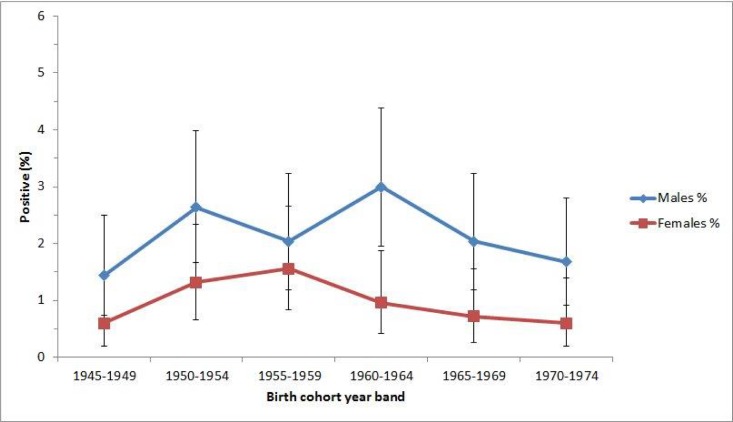
HCV antibody prevalence, by birth cohort year-band and sex.

Of all HCV antibody positive samples, 107/155 (69.03%) were from males, significantly more than from females (p<0.0001) (Table 2). The overall prevalence of HCV antibody in males was 2.14% (95% CI 1.76, 2.58), and in females was 0.96% (95% CI 0.71, 1.27). For both sexes, HCV antibody prevalence was higher in the middle years of the cohort and lowest for the youngest and oldest year-bands (Fig 2). For males, HCV antibody prevalence by year-band of birth ranged from 1.44% (95% CI 0.74, 2.50) for those born between 1945–1949 to 3.00% (95% CI 1.95, 4.39) for those born between 1960–1964. For females, HCV antibody prevalence by year-band of birth ranged from 0.60% (95% CI 0.20, 1.40) for those born between 1945–1949 and 0.60% (95% CI 0.19, 1.39) for those born between 1970–1974 to 1.56% (95% CI 0.83, 2.65) for those born between 1955–1959.

There were no significant differences in the proportion of positive samples from each Ontario region (p = 0.75) (Table 2). HCV seroprevalence varied from 0.88% (95% CI 0.24, 2.24) in the North East region to 2.20% (95% CI 0.60, 5.53) in the North West region. However, both estimates were derived from small numbers of samples, corresponding to Northern Ontario’s small population, resulting in wide CIs around the point estimates. The seroprevalence in Toronto, the largest city in Ontario, was 1.82% (95% CI 1.28, 2.51).

Using a multivariable logistic regression model adjusted for sex, year-band of birth and region, we found that male sex was significantly associated with positive HCV serostatus (OR 2.26, 95% CI 1.60, 3.19) (Table 3). The highest odds of being HCV seropositive were in individuals born between 1950–1954 and 1960–1964. These age-groups were 1.78 times (95% CI 1.00, 3.14) and 1.77 times (95% CI 1.00, 3.13) more likely to be HCV seropositive compared to those born from 1970–1974, although this association did not reach statistical significance. There was no interaction between sex and year-band of birth.

**Table 3:** Characteristics associated with HCV seropositive status in a multivariable logistic regression model, Ontario 2014–2015.

Characteristic		Odds Ratio				
		Crude OR (95% CI)	p-value	Adjusted (95% CI)*		p-value
**Year-band**						
**1945–1949** **1950–1954** **1955–1959** **1960–1964** **1965–1969** **1970–1974**		0.90 (0.46, 1.73) 1.75 (0.99, 3.10) 1.59 (0.89, 2.84) 1.75 (0.99, 3.09) 1.21 (0.66, 2.24) 1.00 (reference)	0.74 0.05 0.12 0.05 0.54 -	0.91 (0.47, 1.75) 1.78 (1.00, 3.14) 1.61 (0.90, 2.87) 1.77 (1.00, 3.13) 1.22 (0.66, 2.25) 1.00 (reference)		0.77 0.05 0.11 0.05 0.53 -
**Sex**						
**Male** **Female**		2.26 (1.60, 3.18) 1.00 (reference)	<0.0001 -	2.26 (1.60, 3.19) 1.00 (reference)		<0.0001 -
**Region**						
**Central East** **Central West** **Eastern** **North East** **North West** **South West** **Toronto**		1.00 (reference) 0.95 (0.58, 1.53) 0.95 (0.55, 1.63) 0.58 (0.21, 1.63) 1.48 (0.53, 4.17) 1.18 (0.70, 1.98) 1.22 (0.78, 1.90)	- 0.82 0.85 0.31 0.46 0.55 0.38	1.00 (reference) 0.95 (0.59, 1.54) 0.95 (0.55, 1.63) 0.58 (0.21, 1.61) 1.44 (0.51, 4.06) 1.17 (0.69, 1.97) 1.24 (0.80, 1.94)		- 0.83 0.84 0.29 0.49 0.56 0.34

*- Adjusted for year-band, sex and region

### Population prevalence estimate

If extrapolated to the Canadian population, the measured prevalence in our study would predict that that there are an estimated 227,203 (95% CI 150,316, 330,208) HCV antibody positive individuals in Canada born between the years 1945 to 1974. Using Remis’ estimates for prevalence outside the 1945–1974 cohort [16], an additional 134,926 individuals are likely to be anti-HCV positive, leading to an overall number of approximately 362,129 infected individuals in Canada. Assuming a spontaneous clearance rate of 26% [22], our data would suggest that 267,975 individuals have chronic HCV in Canada.

## Discussion

To our knowledge, this is the first study in Canada to assess HCV birth-cohort seroprevalence using residual sera from outpatient laboratory testing. Our analysis reveals an elevated prevalence of HCV antibodies in those born between 1950–1964, with a lower but still elevated prevalence in those born between 1965–1969, compared to those born between 1945–1949 and 1970–1974. This is concordant with studies from other regions, particularly the US, where the 1945–1965 birth cohort was found to account for 76.5% of prevalent HCV infections [10]. The anonymized nature of the testing precludes an exploration of risk factors to explain the higher prevalence in this population. However, based on data from Canada and other regions, it likely reflects past injection drug use as well as iatrogenic transmission through transfusion of blood products or medical procedures abroad or in Canada [23]. Interestingly, we observed a dip in HCV antibody prevalence in samples from males born between 1955–1959 compared to other year-bands in the middle of the cohort. Whether this dip truly represents decreased prevalence in this group or is a statistical aberration is unclear, however, the prevalence in this group was still elevated compared to the oldest and youngest cohorts.

Most estimates of Canadian HCV prevalence to date have been based on modeling from literature using estimates of the numbers in specific risk populations and the estimated prevalence in that population [2,16] or disease reporting data, which represent a mix of incidenct and prevalent cases [15,24]. Using the former approach, in a report for the Public Health Agency of Canada, Remis and colleagues estimated an overall HCV antibody prevalence of 0.78%, rising to just over 1% in older adults [16]. Using similar methods with updated data, Trubnikov estimated an HCV antibody prevalence of 0.96% with a chronic HCV prevalence of 0.71%, assuming a 26% spontaneous clearance rate [2]. Trubnikov also applied the ‘back-calculation’ modeling approach, which estimates prevalence based on outcomes of HCV-related disease and the known natural history of infection, and came to an estimate of chronic HCV prevalence (viremic) of 0.64%. Studies in high-risk populations, using both incidence and diagnostic testing data, have also been performed [25–27]. Although very informative, these estimates are not representative of the general population. Lastly, antibody prevalence measures have been derived from cohort studies of healthy populations, such as first time blood donors [28] or other study participants. These studies recruit participants from the general population and often exhibit a participation bias towards healthy individuals, and underrepresentation from high-risk populations. Consequently, although these studies each suggested increased HCV prevalence in our birth cohort of interest, the overall prevalence estimates were imprecise and, perhaps not surprisingly, quite low compared to those presented in this study. For example, the Canadian Health Measures Survey [11] reported an overall HCV seroprevalence of 0.50%, and a seroprevalence of 0.80% for those age 50–79 years.

Although to date, most Canadian HCV prevalence estimates have been lower than those found in our study, our estimates are lower than the estimated 3.25% prevalence in Americans born between 1945–1965 [10], likely due to differences in the distribution of underlying risk factors and social determinants between the two countries.

There are several limitations to our study. Residual sera submitted for diagnostic testing may reflect a selection bias towards individuals with comorbidities, which may include HCV and could thus overestimate the prevalence in the population. Conversely, our estimates may be falsely low due to the fact that high-risk individuals who make up a substantial proportion of individuals with HCV, such as persons who inject drugs or incarcerated populations, are likely underrepresented as they are less likely to access healthcare services. Since serosurveys use de-identified specimens, we have no information on exposures for participants, limiting our ability to further characterize risk factors for HCV infection in this population. Although large urban centres such as the Greater Toronto Area and Ottawa may contain a higher concentration of high-risk groups, these populations were not specifically over-sampled and as such still likely represent a small proportion of the overall cohort, leading to the likelihood that our estimates are lower than the true seroprevalence in Ontario. We also have no information on what proportion of individuals who tested positive are aware of their infection. Due to the potential for degradation of RNA in samples that were not kept frozen, we were not confident that HCV RNA testing would yield accurate results and thus we were not able to determine the number of HCV cases with chronic infection. However, data in other cohorts suggest that the spontaneous clearance rates in the adult population are approximately 26% [22], allowing us to make reliable estimates of the prevalence with chronic infection.

Although the geographically representative sample has likely yielded accurate estimates at the provincial level, the number of specimens submitted from Northern Ontario was small, resulting in wide confidence intervals for prevalence estimates in this region. This may be particularly relevant given the higher Aboriginal population in this region, who have a significantly higher prevalence of HCV than the non-Aboriginal Canadian population [29].

Recently, the Canadian Task Force on Preventative Health Care recommended against birth-cohort screening for HCV due to a lack of high-quality evidence on the effectiveness of screening, high resource implications and financial barriers to accessing treatment [19]. However, the prevalence estimates used in the Task Force recommendations were markedly lower than the ones presented here, and were based on modeling studies rather than population-level seroprevalence data. Our study therefore generates essential data to reframe policy discussions regarding the implementation of population screening strategies for HCV in those born between 1945 and 1974.

This study from a large population-based sample provide robust estimates of HCV prevalence in the population, which are higher than previous estimates from modeling studies, underscoring the value of serosurveys for accurate prevalence data. These results provide updated data to underpin birth cohort screening recommendations for HCV in Canada, in the context of recently available effective direct-acting antiviral drug regimens.
